# Supplementary material for: Galectin-3 is Associated with Cardiovascular Events in Post-Acute Coronary Syndrome Patients with Type-2 Diabetes
Source: J Clin Med. 2020 Apr 13;9(4):1105. doi: 10.3390/jcm9041105 (PMC7230213; doi:10.3390/jcm9041105)
Supplement: Supplementary file 1 [file jcm-09-01105-s001.pdf]

Appendix A

Supplemental Table 1.

Akaike's information criterion (AIC) values for the incidence of acute ischemic events, heart failure or death in non diabetic or diabetic patients adding to the Model 1 the effect of the different biomarkers (NT-proBNP, MCP-1 and Gal-3).

No diabetic Patients	AIC
Model 1	1369.41
Model 1 + NT-proBNP	1365.55
Model 1 + MCP-1	1344.52
Model 1 + NT-proBNP + MCP-1	1339.85
Diabetic Patients	
Model 1	525.59
Model 1 + Gal-3	521.83

Model 1: Adjusted by age, gender, smoking status, hypertension, body mass index, and low-density lipoprotein, high-density lipoprotein, and triglyceride plasma levels, history of cerebrovascular events, ejection fraction <40%, or atrial fibrillation; glomerular filtration rate assessed by Chronic Kidney Disease Epidemiology Collaboration method <60, high-sensitivity C-reactive protein, therapy with acetylsalicylic acid, antiP2Y12, anticoagulants, statins, angiotensin-converting enzyme inhibitors, angiotensin receptor blockers (ARB), anti-aldosterone, β-blockers, nitrates and/or nitroglycerin, diuretic and, type of last acute coronary event or existence of complete revascularization at the event. In diabetic patients, model 1 also included therapy with insulin or oral antidiabetic drugs.

Supplemental Table 2.

Cox proportional hazards model for the incidence of acute ischemic events, heart failure or death in diabetic-hypertensive patients

Variable	Model 1	Model 2
	HR (95% CI)	HR (95% CI)
Age, years	1.04 (0.99-1.08)	1.04 (1.00-1.08)
Sex, male	0.34 (0.14-0.84)	0.42 (0.17-1.08)
Smoker, yes	2.43 (0.90-6.58)	3.30 (1.10-9.90)
Body mass index, kg/m^2	0.99 (0.97-1.01)	0.99 (0.97-1.01)
History of CVE, yes	7.12 (1.58-32.1)	6.39 (1.28-32.0)
Ejection fraction <40%, yes	0.58 (0.23-1.46)	0.62 (0.24-1.59)
Atrial Fibrillation, yes	2.76 (0.79-9.61)	3.01 (0.80-11.3)
Acute myocardial infarction, yes	1.31 (0.61-2.85)	1.24 (0.57-2.70)
Complete Revascularization	0.87 (0.43-1.76)	1.02 (0.50-2.08)

**LDL-c, mg/dL**	**1.00 (0.98-1.01)**	**1.00 (0.98-1.01)**
**HDL-c, mg/dL**	**1.02 (0.98-1.06)**	**1.02 (0.98-1.06)**
**Triglycerides, mg/dL**	**1.00 (1.00-1.01)**	**1.00 (1.00-1.01)**
**CKD-EPI <60 mL/min/1.73 m^2**	**0.91 (0.41-2.01)**	**0.82 (0.36-1.89)**
**Acetylsalicylic acid, yes**	**0.65 (0.14-2.98)**	**0.88 (0.19-4.14)**
**AntiP2Y12, yes**	**0.42 (0.19-0.93)**	**0.44 (0.19-1.01)**
**Anticoagulants, yes**	**2.45 (0.59-10.2)**	**2.54 (0.57-11.3)**
**Statins, yes**	**0.31 (0.10-0.94)**	**0.32 (0.09-1.13)**
**ACE inhibitors, yes**	**0.48 (0.21-1.14)**	**0.50 (0.20-1.24)**
**ARB, yes**	**0.85 (0.32-2.23)**	**0.72 (0.25-2.03)**
**Anti-aldosterone, yes**	**2.64 (0.92-7.55)**	**2.15 (0.71-6.50)**
**β-Blockers, yes**	**1.14 (0.51-2.54)**	**1.05 (0.47-2.34)**
**Nitrates, yes**	**1.77 (0.80-3.94)**	**1.68 (0.75-3.78)**
**Diuretics, yes**	**1.07 (0.51-2.22)**	**0.94 (0.42-2.10)**
**Insulin, yes**	**2.15 (1.05-4.42)**	**2.26 (1.03-4.95)**
**Oral antidiabetic drugs, yes**	**1.00 (0.46-2.16)**	**1.07 (0.47-2.46)**
**Hs-CRP, mg/L**	**0.98 (0.94-1.02)**	**0.97 (0.92-1.02)**
**NT-proBNP, 1-SD**	**-------------**	**1.01 (0.76-1.36)**
**MCP-1, 1-SD**	**-------------**	**0.90 (0.57-1.42)**
**Gal-3, 1-SD**	**-------------**	**1.78 (1.19-2.68)**
**Hs-cTnT, 1-SD**	**-------------**	**0.93 (0.78-1.10)**

Model 1: Adjusted by age, gender, smoking status, hypertension, body mass index, and low-density lipoprotein, high-density lipoprotein, and triglyceride plasma levels, history of cerebrovascular events, ejection fraction <40%, or atrial fibrillation; glomerular filtration rate assessed by Chronic Kidney Disease Epidemiology Collaboration method <60, high-sensitivity C-reactive protein, therapy with acetylsalicylic acid, antiP2Y12, anticoagulants, statins, angiotensin-converting enzyme inhibitors, angiotensin receptor blockers (ARB), anti-aldosterone, β-blockers, nitrates and/or nitroglycerin, diuretic and, type of last acute coronary event or existence of complete revascularization at the event. In diabetic patients, model 1 also included therapy with insulin or oral antidiabetic drugs.

Model 2: risk adjusted for factors in model 1 and NT-proBNP, MCP-1, galectin-3 and Hs-cTnT.
